# Comparison between the antiemetic effects of palonosetron and granisetron in breast cancer patients treated with anthracycline-based regimens

**DOI:** 10.3892/ol.2014.2640

**Published:** 2014-10-24

**Authors:** HIDEYUKI OHZAWA, ATSUSHI MIKI, YASUO HOZUMI, CHIEKO MIYAZAKI, YUKA SAGARA, YUMIKO TANAKA, SATOMI SHIBA, HIROMI JOUTOKU, MASAKO SAKURAGI, MEGUMI TAKEHARA, YASUNARU SAKUMA, WATARU NISHIMURA, HIROFUMI FUJII, YOSHIKAZU YASUDA

**Affiliations:** 1 Department of Breast Surgery, Jichi Medical University, Shimotsuke City, Tochigi 329-0498, Japan; 2 Department of Gastrointestinal Surgery, Jichi Medical University, Shimotsuke City, Tochigi 329-0498, Japan; 3 Department of Metabolic Disorder, Diabetes Research Center, National Center for Global Health and Medicine, Shinjuku-ku, Tokyo 162-8655, Japan; 4 Department of Clinical Oncology, Jichi Medical University, Shimotsuke City, Tochigi 329-0498, Japan

**Keywords:** palonosetron, granisetron, antiemetic therapy

## Abstract

Chemotherapy-induced nausea and vomiting is a serious adverse side-effect of anthracycline-based chemotherapy regimens, in patients with breast cancer. A combination of three drugs, 5-hydroxytryptamine (5-HT_3_) receptor antagonist, aprepitant and dexamethasone, is recommended for antiemetic therapy. Palonosetron (PALO), a novel 5-HT_3_ receptor antagonist has been identified to be effective against delayed nausea and vomiting. In this study, the results of PALO for patients who received anthracycline-based chemotherapy were compared with that of granisetron (GRA) using a crossover study design. This study evaluated the efficacy of antiemetics in the first cycle of chemotherapy, as well as the second and third cycles. A total of 21 patients and 19 patients were assigned to PALO and GRA treatment groups during the first cycle of chemotherapy, respectively. The patients switched to the other antiemetic drug for the second chemotherapy cycle (PALO followed by GRA or GRA followed by PALO). The patients could select PALO or GRA antiemetics for the third cycle, according to their preference. A total of 21 patients selected PALO and 18 patients selected GRA in the third cycle, and one patient was withdrawn from the study as their third cycle questionnaire was not obtained. No significant differences between PALO and GRA were identified in first and second cycles. However, during the third cycle, a significant difference was observed in acute-phase complete control of emetic events between the PALO and GRA groups, which was defined as no emetic episode, no additional antiemetic treatment and no more than mild nausea, between PALO and GRA. These results demonstrated that changing antiemetics may affect the efficacy of antiemetics. This study indicates that alteration of antiemetic regimens, including drug combination and order, may improve the efficacy of antiemetic treatment.

## Introduction

Combination chemotherapy regimens for breast cancer, which include anthracycline drugs and cyclophosphamide [doxorubicin plus cyclophosphamide (AC); epirubicin plus cyclophosphamide (EC); and fluorouracil, epirubicin plus cyclophosphamide (FEC)], are classified as exhibiting a high risk of emesis by the National Comprehensive Cancer Network in 2012 and American Society of Clinical Oncology guidelines (1,2). It is recommended in these guidelines to use a combination of three drugs [5-hydroxytryptamine (5-HT_3_) receptor antagonist, aprepitant (APR) and dexamethasone (DEX)] for antiemetic treatment (1,2). Recently, a novel 5-HT_3_ receptor antagonist, palonosetron (PALO), has been identified. PALO has demonstrated effectiveness against delayed emetic events (3–5). PALO and APR excel in the prevention of delayed nausea and vomiting. However, no studies regarding the comparative efficacy of PALO and the conventional 5-HT_3_ receptor antagonists used in combination with APR have been reported.

In the present study, the efficacy of the novel 5-HT_3_ receptor antagonist, PALO, was compared with that of the conventional drug granisetron (GRA) for the antiemetic treatment of breast cancer patients treated with highly emetic therapeutic regimens that involved anthracyclines and cyclophosphamide. A crossover administration method was used, with the administration of two cycles of antiemetic agents. Furthermore, no studies have investigated the efficacy of such drugs, following the second cycle and, thus, in the present study, the efficacy of the drugs were also evaluated following the second cycle.

## Materials and methods

### Patients

This study was approved by the ethics committee of Jichi Medical University (B10–68; Tochigi, Japan) and written informed consent was obtained from all patients. This investigation was a prospective, stratified randomization, non-blinded, crossover comparative study. Eligible patients were females (≥20 years; age range, 35–75 years) with histologically confirmed breast cancer, who were scheduled to receive chemotherapy including anthracycline drugs and cyclophosphamide at the Department of Breast Surgery, Jichi Medical University Hospital. Prior to the first cycle of chemotherapy, 40 patients were assigned to two groups treated with PALO or GRA first. The group assignment was performed by simple randomization using a table of random numbers and patients were informed of which group they were assigned.

### Treatment and evaluation

**Figure 1:**
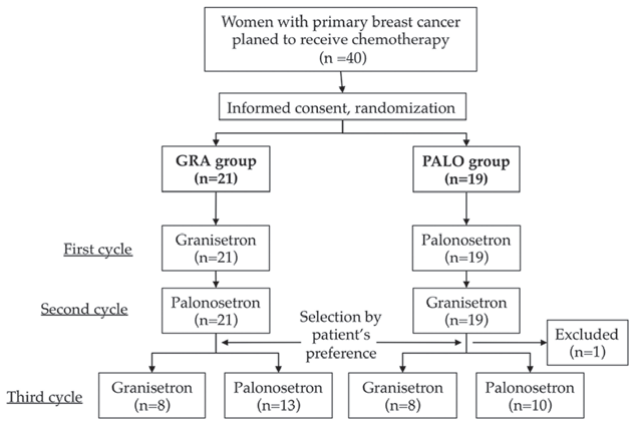
Study flowchart showing the treatment regimens of the patients and the selection of antiemetic drugs for the third cycle. GRA, granisetron; PALO, palonosetron.

Chemotherapy was administered every three weeks as follows: AC treatment, adriamycin (60 mg/m^2^) and cyclophosphamide (600 mg/m^2^); EC treatment, epirubicin (90 mg/m^2^) and cyclophosphamide (600 mg/m^2^); FEC treatment, 5-fluorouracil (500 mg/m^2^), epirubicin (100 mg/m^2^) and cyclophosphamide (500 mg/m^2^). Patients were assigned to the PALO or GRA group in the first cycle, as described above. For the second cycle of treatment, patients switched to the other medication (GRA followed by PALO or PALO followed by GRA). Prior to beginning the third cycle, patients selected GRA or PALO based on their preferences, and chemotherapy was continued (Fig. 1).

As an antiemetic treatment prior to chemotherapy, APR (125 mg) was orally administered 1 h prior to treatment, and PALO (0.75 mg) or GRA (3 mg) was administered in addition to DEX (13.2 mg) 30 min prior to chemotherapy by intravenous infusion. Chemotherapy was then administered. APR (80 mg) was orally administered on days two and three following chemotherapy, and DEX (8 mg) was administered orally on days two, three and four following chemotherapy (Table I). When additional antiemetic treatment was required, metoclopramide was administered orally, or additional APR was administered orally on the fourth and fifth days following chemotherapy.

To evaluate instances of nausea and vomiting, patients were asked to complete a questionnaire on antiemetics, as well as a patient log. Adverse effects and blood tests were evaluated prior to each cycle of chemotherapy, and attending physicians decided whether to continue chemotherapy in accordance with the criteria used for usual care.

The antiemetic efficacy of the drugs was evaluated until the third cycle of chemotherapy was completed. The efficacy was rated on the basis of complete control of acute and delayed vomiting (complete response; CR) and complete control of emetic events (complete control; CC). CR was defined as no emetic episode and no additional antiemetic treatment. CC was defined as no emetic episode, no additional antiemetic treatment, and no more than mild nausea.

Participation in the study was discontinued for the following reasons: If general and disease status became worse than that prior to study participation; the attending physician judged the continuation of chemotherapy to be difficult; the patient requested to withdraw from the study or withdrew consent; or the circumstances of the patient made continuation impossible.

### Statistical analysis

Statistical analyses were performed using JMP statistical software, version 10 (SAS, Institute Inc., Cary, NC, USA). The χ^2^ test was used for statistical analysis, and P<0.05 was considered to indicate a statistically significant difference.

## Results

### Patients

A total of 19 patients received PALO first and 21 patients received GRA first. Among the PALO-first group, the third-cycle questionnaire was not obtained from one patient and, thus, this case was withdrawn from analysis of the third cycle (Fig. 1). The median ages of the patients in the GRA-first group were 53 years (range, 40–71 years) and 53 years (range, 35–75 years) in the GRA and PALO-first groups, respectively (Table II).

### Treatment efficacy in the first cycle

**Figure 2:**
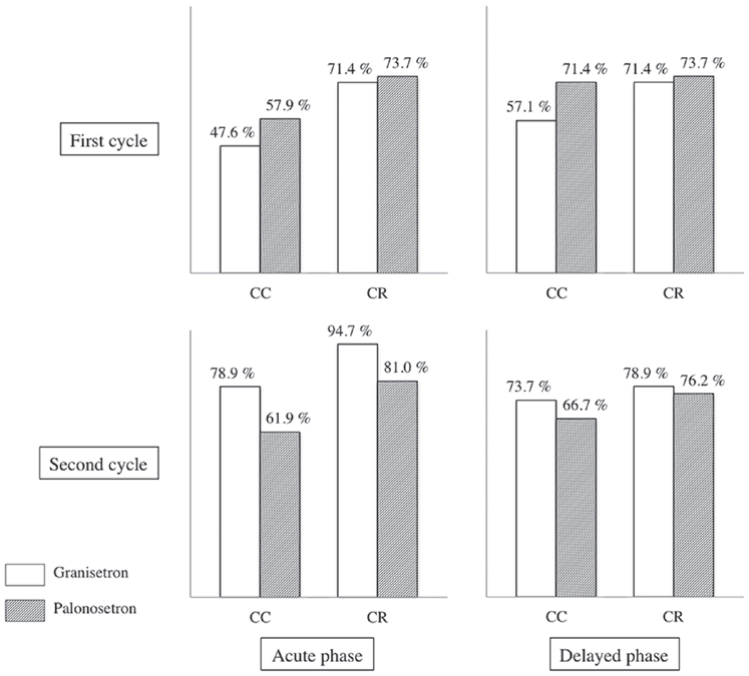
Antiemetic efficacy of granisetron and palonosetron in the first and second cycles of chemotherapy. The efficacy was evaluated by CC rate and CR rate. In the first cycle, granisetron and palonosetron were administered to GRA-first group and PALO-first group, respectively. In the second cycle, the antiemetics were switched. Therefore, granisetron and palonosetron were administered to PALO-first group and GRA-first group, respectively. No significant differences in CC or CR were identified between granisetron and palonosetron. CC, complete control (no emetic episode, no additional antiemetic treatment and no more than mild nausea); CR, complete response (no emetic episode and no additional antiemetic treatment); GRA, granisetron; PALO, palonosetron.

In the first cycle, acute-phase CC was observed in 47.6% and 57.9% of patients of the GRA-first group and PALO-first groups, respectively (P=0.515) (Fig. 2). Acute-phase CR was observed in 71.4 and 73.7% patients of the GRA-first and PALO-first groups, respectively (P=0.873). Delayed-phase CC was observed in 57.1 and 71.4% of patients in the GRA-first and PALO-first groups, respectively (P=0.461). Delayed-phase CR was observed in 71.4 and 73.7% of patients in the GRA-first and PALO-first groups, respectively (P=0.873).

### Treatment efficacy in the second cycle

In the second cycle, acute-phase CC of emetic events was observed in 61.9 and 78.9% of patients in the GRA-first and PALO-first groups, respectively (P=0.240) (Fig. 2). Acute-phase CR was observed in 81.0 and 94.7% of patients in the GRA-first and PALO-first groups, respectively (P=0.019). Delayed-phase CC was observed in 66.7 and 73.7% of patients in the GRA-first and PALO-first groups, respectively (P=0.628). Delayed-phase CR was observed in 76.2 and 78.9% of patients in the GRA-first and PALO-first groups, respectively (P=0.834).

### Treatment efficacy in the third cycle

**Figure 3:**
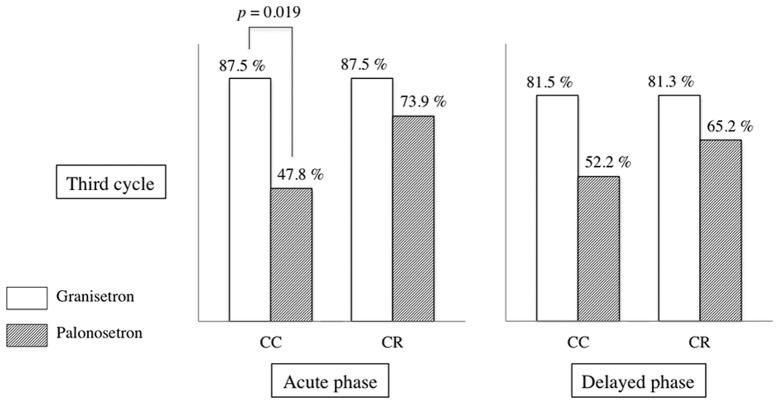
Antiemetic efficacy of granisetron and palonosetron in the third cycle of chemotherapy. In the third cycle, patients selected granisetron or palonosetron according to their preference. A total of 18 patients selected granisetron and 21 patients selected palonosetron. A significant difference was identified in the acute phase CC of emetic events between the granisetron and palonosetron treatment groups. CC, complete control (no emetic episode, no additional antiemetic treatment, and no more than mild nausea); CR, complete response (no emetic episode and no additional antiemetic treatment).

In the third cycle, a total of 46.2% of the patients (18/39) selected GRA and 53.8% (21/39) selected PALO. In the third cycle, a significant difference in acute-phase CC of emetic events was identified between PALO and GRA treatment groups. Acute-phase CC was observed in 87.5 and 47.8% of patients in the GRA-selection and PALO-selection groups, respectively (P=0.011) (Fig. 3). Acute-phase CR was observed in 87.5 and 73.9% of patients in the GRA-selection and PALO-selection groups, respectively (P=0.301). Delayed-phase CC was observed in 81.3 and 52.2% of patients in the GRA-selection and PALO-selection groups, respectively (P=0.273). Delayed-phase CR was observed in 81.3 and 65.2% of patients in the GRA-selection and PALO-selection groups, respectively (P=0.273).

## Discussion

To the best of our knowledge, this was the first study to use crossover administration of first- and second-cycle antiemetic agents in association with chemotherapy for breast cancer to compare the efficacy of these agents. The majority of previous studies have evaluated the efficacy of antiemetic agents following the first cycle of chemotherapy, and few studies regarding the efficacy from the second cycle onwards have been performed. In the current study, the efficacy of the drugs following the second and third cycles was also evaluated. In the GRA- and PALO-first groups, the prevalence of acute-phase CC increased between the first to second cycle. In addition, the prevalence of acute-phase CC in the third cycle decreased in patients who selected PALO treatment. This result indicated that an order effect was exhibited in PALO followed by GRA and GRA followed by PALO patients, and a carry over effect was exhibited in PALO followed by PALO patients. Considering these effects, antiemetic treatment in breast cancer chemotherapy requires a refined administration design for optimal efficacy.

As side effects of breast cancer chemotherapy, nausea and vomiting are often problematic (1). Emetic events lead to a decrease in appetite and body weight, reducing the quality of life (1,7). In addition, the dose of chemotherapy is considered to have an impact on prognosis. Identifying ways to complete chemotherapy with fewer side effects is important to improve the treatment outcome. AC, EC and FEC, representative chemotherapy regimens for perioperative early-stage breast cancer, are anthracycline-based regimens with a high emetic risk, which require effective prevention of chemotherapy-induced nausea and vomiting (CINV). Treatment-related factors and patient-related factors are associated with CINV. Treatment-related factors include the type and dose of anti-cancer drugs and patient-related factors include women aged <50 years with no history of pregnancy and with no history of alcohol consumption (8–10).

To date, APR and PALO have been reported to exhibit effective delayed antiemetic effects (1,3). However, the efficacy of these agents in combination has not been investigated. APR primarily affects the vomiting reaction pathway in the central nervous system (CNS) and has selective neurokinin-1 (NK-1) receptor antagonist actions (1). It is hypothesized to prevent and control acute and delayed nausea and vomiting. CINV develops when the vomiting center in the medulla oblongata receives a stimulus. The two main pathways for this stimulus have been hypothesized to be the CNS pathway and peripheral pathways. NK-1 receptors, which bind substance P and 5-HT_3_ receptors that bind serotonin are known to be involved in this process. Substance P is hypothesized to be dominant in the CNS pathway and 5-HT_3_ is considered to be dominant in the peripheral pathway (11,12). For acute-phase emesis, the two receptors are associated with vomiting. However, in the case of delayed emesis, the impact of substance P is considered to become dominant (12), which is regarded to be a cause for limited antiemetic action of 5-HT_3_ receptor antagonists for delayed vomiting.

PALO and GRA are 5-HT_3_ receptor antagonist antiemetic agents. PALO differs from conventional drugs as it has an extremely long half-life in the blood (~40 h), as well as high affinity and selectivity for 5-HT_3_ receptors. Thus, it has been identified to be efficacious for the treatment of delayed nausea and vomiting, which occur ≥24 h following chemotherapy. The delayed effects of PALO are considered to be a result of its slow release after binding to the receptors, with a reported continuation of receptor inhibition of >96 h. It has also been reported that PALO induces internalization of the receptor on the cell surface, causing allosteric downregulation (4,13,14) and that PALO controls substance P independently of serotonin (15). In the present study, no significant difference was identified between delayed vomiting in the PALO-first and GRA-first groups. However, the efficacy of 5-HT_3_ receptor antagonist antiemetic drugs against delayed vomiting may be masked by the administration of APR.

A limitation of the present study was the small patient cohort. Although, by employing the prospective study design, the cohort was considered to be sufficient. The evaluation of vomiting and nausea is difficult; however, the evaluation of CC and CR was possible via the use of patient logs and survey questionnaires. It has been reported that psychological elements also have an impact on nausea. Psychological aspects were not considered in this study; however, these factors may have exhibited an effect on drug selection for the third cycle or order effects. A crossover treatment was used in this study. However, each drug had a short half-life and, thus, in terms of the three-week drug intervals, it is hypothesized that the effects of the drugs on the next cycle administered prior to the start of the cycle were small.

In conclusion, the GRA-selection group in the third cycle exhibited a significant difference in acute-phase CC and CR when compared with the PALO group, and the effect of vomit control was observed. No significant difference between delayed-phase CC and CR was identified, and APR and PALO did not affect each other. These results differ from those reported previously. However, the effects of PALO may have been inhibited due to the presence of APR. Hence, considering order or carry over effects, a novel three-drug antiemetic regimen involving PALO in the first cycle followed by GRA in later cycles may present a novel treatment for breast cancer patients.

## Figures and Tables

**Table I:** Schedule for administration of antiemetic drugs.

Antiemetic regimen	Drug (administration method)		Day 1	Day 2	Day 3	Day 4
Palonosetron	Palonosetron	i.v.	0.75 mg			
	Aprepitant	p.o.	125 mg	80 mg	80 mg	
	Dexamethasone	i.v.	13.2 mg			
	Dexamethasone	p.o.		8 mg	8 mg	8 mg
Granisetron	Granisetron	i.v.	3 mg			
	Aprepitant	p.o.	125 mg	80 mg	80 mg	
	Dexamethasone	i.v.	13.2 mg			
	Dexamethasone	p.o.		8 mg	8 mg	8 mg

i.v, intravenous administration; p.o., oral administration.

**Table II:** Patients characteristics.

Parameter	GRA group (n=21)	PALO group (n=19)
Median age, years (range)	53 (40–71)	53 (35–75)
Menopause status, n (%)		
Premenopause	10 (47.6)	10 (52.6)
Postmenopause	11 (52.4)	9 (47.4)
ECOG performance status, n (%) (6)		
0	21 (100.0)	19 (100.0)
Chemotherapy regimen, n (%)		
FEC	11 (52.4)	12 (63.2)
AC/EC	10 (47.6)	7 (36.8)
Timing of chemotherapy, n (%)		
Neoadjuvant	19 (90.5)	16 (84.2)
Adjuvant	2 (9.5)	3 (15.8)

GRA, granisetron; PALO, palonosetron; ECOG, Eastern Cooperative Oncology Group; FEC, fluorouracil, epirubicin and cyclophosphamide; AC, doxorubicin and cyclophosphamide; EC, epirubicin and cyclophosphamide.
